# A Benchmark Dataset of Agricultural Weather Stations over the Contiguous United States for Evapotranspiration Applications

**DOI:** 10.1038/s41597-026-07819-7

**Published:** 2026-08-12

**Authors:** Christian Dunkerly, John M. Volk, Sayantan Majumdar, Justin L. Huntington, Richard G. Allen, Christopher Pearson, Yeonuk Kim, Charles G. Morton, Blake A. Minor, Peter ReVelle, Ayse Kilic, Forrest Melton, Adam J. Purdy, Todd G. Caldwell

**Affiliations:** 1 Desert Research Institute, Division of Hydrologic Sciences, Reno, Nevada USA; 2 University of Idaho, Kimberly, Idaho USA; 3 University of Nebraska-Lincoln, School of Natural Resources, Lincoln, Nebraska USA; 4 NASA Ames Research Center, Moffett Field, California USA; 5 California State University, Monterey Bay, Seaside, California USA

## Abstract

Agricultural weather data are fundamental for the accurate estimate of evapotranspiration (ET), irrigation scheduling, and water-use accounting. In particular, reference ET provides a standardized atmospheric demand for water loss from a hypothetical well-watered grass (ETo) or alfalfa (ETr); however, weather stations may not adequately represent such climatic conditions. Weather data commonly contain errors from poor siting, sensor drift, and network management deficiencies. No standardized dataset exists over the contiguous United States (CONUS). Systematic errors affect ETo/ETr calculations and derived products. Notably, satellite-based platforms like OpenET require agricultural weather data to bias correct gridded reference ET to interpolate between satellite overpasses. CONUS-AgWeather is a benchmark dataset of daily agricultural weather data (precipitation, solar radiation, air temperature, humidity, wind speed, ETo, ETr) from 793 stations. This dataset contains 4,191,808 days (11,484 station-years, 1981–2020) and was produced through standardized and systematic quality control procedures and open-source software packages for time series inspection, outlier detection, corrections, and ETo/ETr calculations. CONUS-AgWeather is intended primarily to support OpenET in the Western U.S. but has broader applications.

## Background & Summary

The sustainable management of water resources through measurement and optimization of agricultural water use and crop productivity increasingly requires accurate and timely weather information. Weather data, such as solar radiation, air temperature, humidity, wind speed, and precipitation, are fundamental inputs for a multitude of agricultural applications. These data are necessary for calculating reference evapotranspiration (ET), a key determinant of crop water requirements, which directly informs irrigation system design, scheduling, and water allocation decisions^1,2^. Beyond reference ET, weather data can be directly used to assess land surface–atmospheric boundary layer feedbacks and regional actual ET^3,4^. Furthermore, high-quality weather data serve as essential direct input or ground-truth for calibration and validation of hydrological models^5^, gridded weather datasets^6–8^, and satellite-based ET products used for crop water use monitoring and reporting^9–18^.

Weather conditions, such as solar radiation, temperature, humidity, and wind speed, are closely linked to land surface conditions. For instance, the air above irrigated croplands is typically cooler and more humid than surrounding areas because a larger portion of net radiation is consumed by ET rather than surface heating^19^. Consequently, weather stations located within croplands provide critical information for accurately characterizing agricultural weather conditions and total evaporative demand. Despite their importance, no unified national-scale agricultural weather station network or dataset exists in the United States (U.S.), stations are sparse, and public data access is limited when compared to the assortment of National Weather Service (NWS) stations located within towns, cities, roadways, and airport locations. NWS stations typically collect select weather data of air temperature and precipitation, with airport locations also collecting humidity and wind speed often at heights of 10 m or greater. Solar radiation measurements are often limited to state mesonets only. Agricultural weather stations typically measure the full suite of meteorological variables required to compute physically based evaporative demand or standardized reference ET for short grass (ETo) or tall alfalfa (ETr). However, fragmented funding and limited operations and maintenance often limit rigorous quality assurance, resulting in datasets that contain random and systemic errors. Sensor degradation over time, calibration drift, physical obstructions, inadequate maintenance, communication errors, and non-ideal station siting (e.g., insufficient fetch or deviation from well-watered conditions) can all introduce errors and biases into the observational record^20–22^. If unaddressed, these data quality issues can lead to erroneously high calculations of crop water requirements, ET, flawed irrigation project design, irrigation scheduling, and ultimately, suboptimal crop and water resource management^20,21^.

To address these challenges, observational weather datasets should be subject to robust quality control (QC) procedures before use. Such procedures aim to systematically identify and correct or remove erroneous data, thereby enhancing the overall integrity and reliability of the dataset. While various QC methodologies exist, the development and application of a consistent, transparent, and accessible framework for QC of daily agricultural weather data has been a persistent need. The open-source *agweather-qaqc* Python package^23^ was developed to meet this need, offering a command-line interface (CLI) tool that facilitates reading, visualization, and comprehensive QC of daily weather observations from diverse sources, followed by the calculation of reference ET using standardized methods and guidelines by the American Society of Civil Engineers and Environmental and Water resources Institute (ASCE-EWRI)^24^.

## Data Overview

**Fig. 1:**
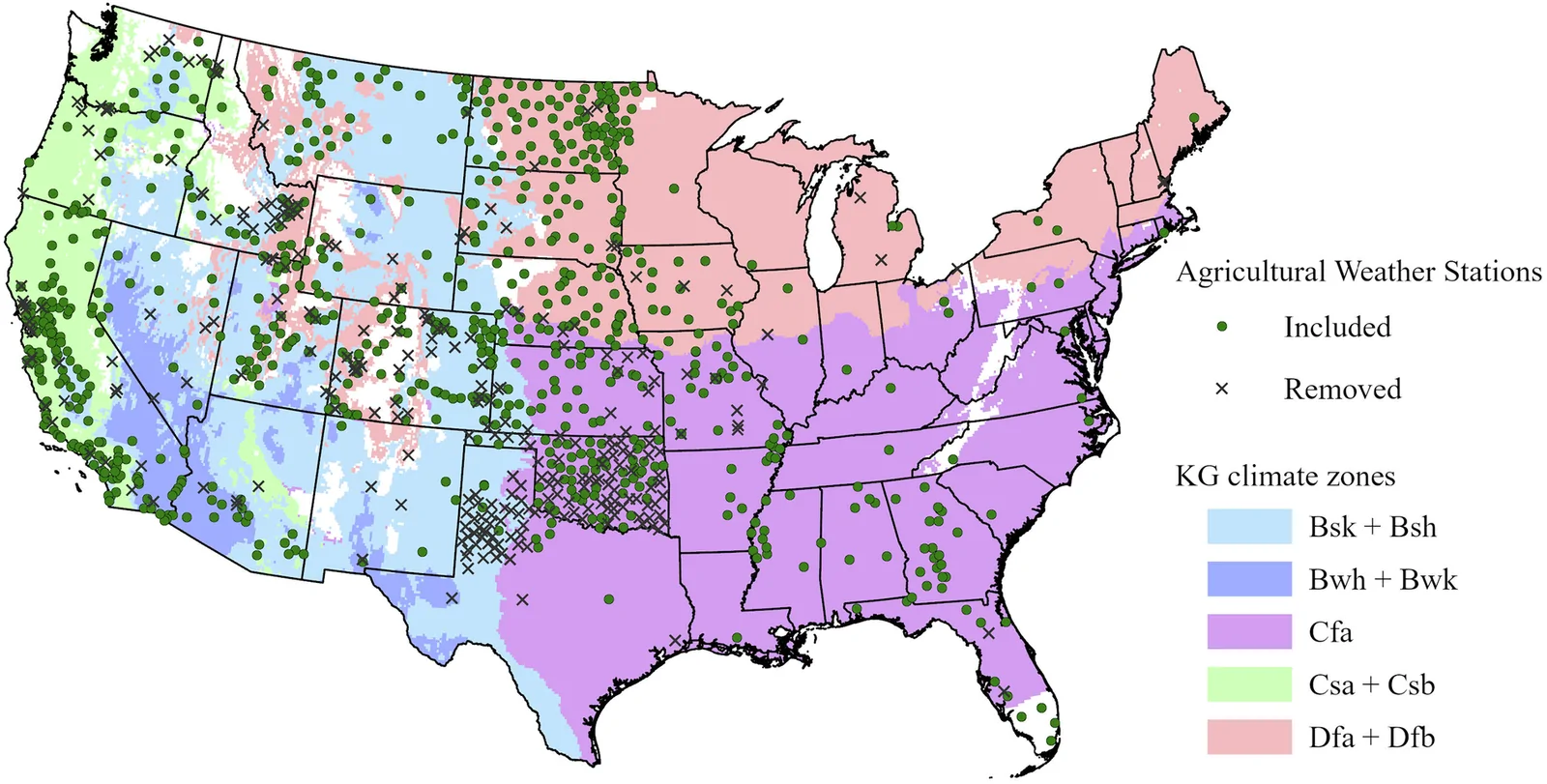
Map showing the distribution of the initial 1,078 agricultural weather stations, of which 793 were included and 285 were removed after site evaluation and data QC, overlaid on major Köppen-Geiger (KG) climate zones^25^: cold and hot semi-arid steppe (Bsk + Bsh); hot and cold desert (Bwh + Bwk); humid subtropical (Cfa); hot- and warm-summer Mediterranean (Csa + Csb); and hot- and warm-summer humid continental (Dfa + Dfb). Here, stations over white no-data areas are in other KG climate zones. The CONUS-AgWeather dataset includes a total of 4,191,808 days (11,484 years) of data spanning May 21, 1981, to December 31, 2020, with record lengths varying by each station.

CONUS-AgWeather is a high-quality benchmark dataset of daily weather and reference ET variables, compiled from 793 agricultural weather stations from 25 networks across the contiguous U.S. (CONUS) (Fig. 1). The dataset includes precipitation, solar radiation, air temperature, humidity, wind speed, ETo, and ETr, with station records screened for suitability for agricultural reference ET applications. CONUS-AgWeather was initially developed to support the OpenET project with a particular focus on the Western U.S., but is intended for broader use in ET, agricultural water management, and meteorological applications.

**Fig. 2:**
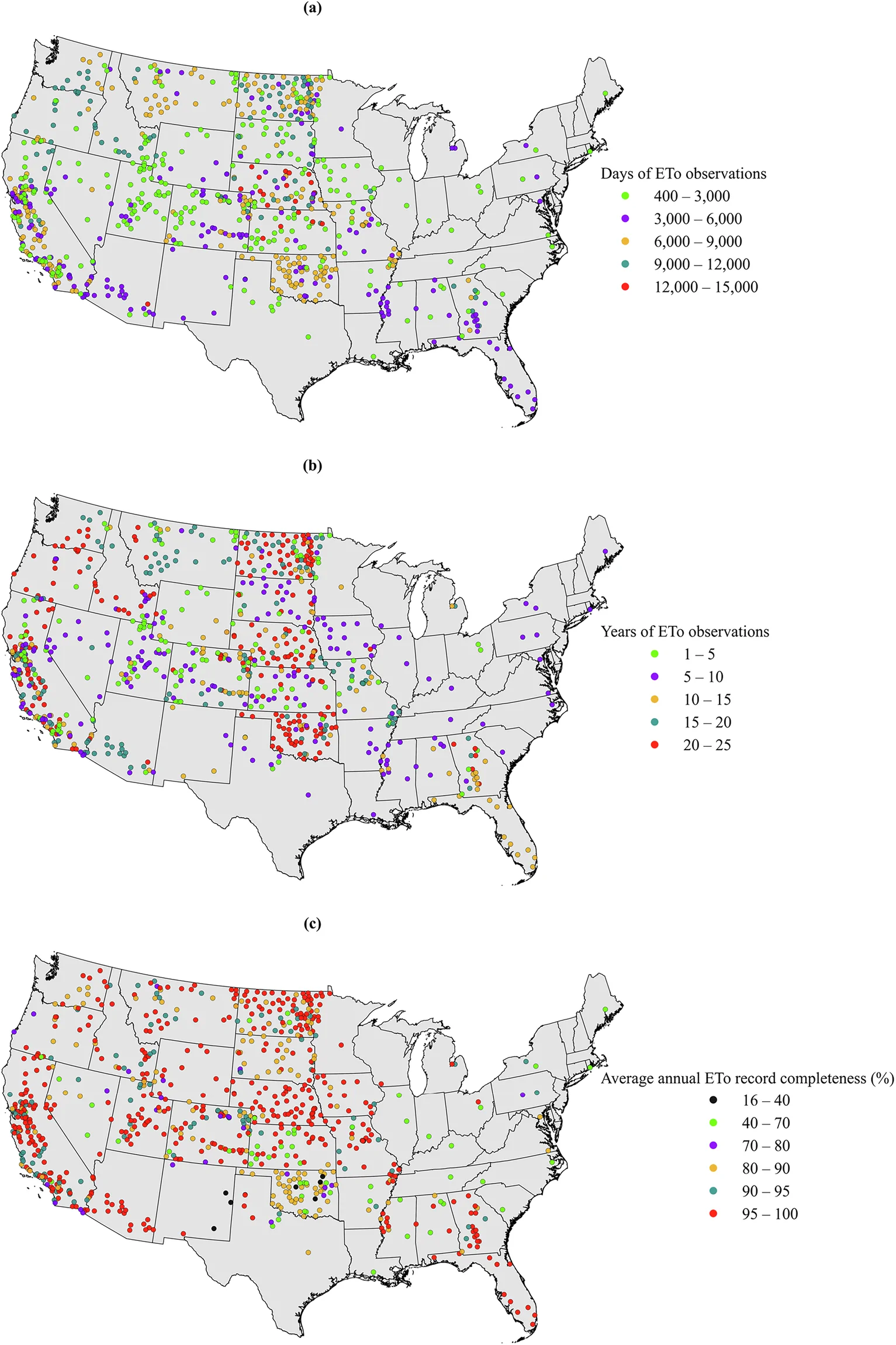
The complete, quality-controlled CONUS-AgWeather station locations showing **(a)** the number of grass reference ET (ETo) observations, **(b)** years of ETo observations, and **(c)** the average annual ETo record completeness for each station. This dataset includes 793 stations with a total of 11,484 years, i.e., 4,191,808 days of valid weather data across the CONUS, spanning from May 21, 1981, to December 31, 2020 (record lengths vary by station). Corresponding maps for all CONUS-AgWeather variables are available on Zenodo (10.5281/zenodo.18122156).

Figure 1 illustrates stations that were included and removed after the QC process and visual site inspections, along with the major Köppen-Geiger (KG) climate zones^25^. Figure 2a shows included stations, colored by the number of valid daily ETo observations for each station after data QC. In addition, the number of ETo years (calculated as the number of ETo observation days for each station divided by 365) and the average annual station record completeness of the available ETo data are highlighted in Fig. 2b and Fig. 2c, respectively. Here, the average annual record completeness (expressed in percentage) for an individual station is defined as the ratio of the number of available ETo observations to the total number of days the station was active within that calendar year. This method normalizes for partial years at the beginning or end of the record by using the actual period of record as the denominator rather than a fixed 365/366-day calendar year. Note, there are two California Irrigation Management Information System (CIMIS)^26^ stations (currently inactive) located in Mexicali, Mexico and San Luis Río Colorado, Mexico that were included given close proximity to the U.S. border and quality long-term records.

## Methods

Development of the CONUS-AgWeather dataset began with the acquisition of raw weather data from 1,078 weather stations illustrated in Fig. 1. Raw weather data underwent extensive QC data processing using the *agweather-qaqc* Python package^23^, with visual inspection of data and site conditions as described below, resulting in 793 station datasets illustrated in Fig. 1.

### Data sources

Agricultural weather station data used in the development of the CONUS-AgWeather dataset were sourced from 25 networks operating throughout the CONUS. These include the U.S. Bureau of Reclamation’s AgriMet Network, Pacific Northwest and Great Plains regions^27^, Arizona Meteorological Network (AZMET)^28^, CIMIS^26^, Colorado Agricultural and Meteorological Network (CoAgMET)^29^, Enviroweather Network^30^, Florida Automated Weather Network (FAWN)^31^, Georgia Automated Environmental Monitoring Network (GAEMN)^32^, High Plains Regional Climate Center (HPRCC)^33^, Missouri Mesonet^34^, North Dakota Agricultural Weather Network (NDAWN)^35^, Nebraska Mesonet^36^, Nevada Integrated Climate and Evapotranspiration Network (NICE Net)^37^, Oklahoma Mesonet^38^, U.S. Department of Agriculture (USDA) Soil Climate Analysis Network (SCAN)^39^, South Dakota Mesonet^40^, National Oceanic and Atmospheric Administration’s United States Climate Reference Network (USCRN)^41^, West Texas Mesonet^42^, Western Regional Climate Center (WRCC)^43^, New Mexico’s ZiaMet^44^, Utah Climate Center (UCC)^45^, USDA Agricultural Research Service (USDA-ARS)^46^, Wyoming Agricultural and Climate Network (WACNet)^47^, Iowa Environmental Mesonet^48^, and Kansas Mesonet^49^. Data were primarily acquired at a daily temporal resolution. If data were acquired at higher temporal resolution (e.g., hourly), data were resampled to daily timesteps following standardized methods prior to ingestion into the QC process^24^. Table 1A shows the number of stations present in the initial dataset (1,078) and the number of stations that were included (793) in the final CONUS-AgWeather dataset after the QC process. Documentation on data access and restrictions for each network are reported in Table 1B.

**Table 1:** A Network information for agricultural weather stations present in the initial dataset (1,078 stations) and the final CONUS-AgWeather dataset (793 stations). B. Data access and use restrictions for the networks included in the dataset.

Network	Initial Number of Stations	Number of Stations Removed	Number of Stations Included	Access Date (yyyy-mm-dd)
AgriMet, Columbia-Pacific Northwest Region^27^	132	45	87	2021-01-10
AgriMet, Missouri Basin Region and Arkansas-Rio Grande-Texas Gulf Regions^27^	25	0	25	2020-02-14
AZMET^28^	29	4	25	2021-01-15
CIMIS^26^	161	23	138	2019-06-10
CoAgMET	101	33	68	2021-01-09
GAEMN^32^	19	0	19	2020-04-17
HPRCC^†,^^33^	249	33	216	2020-07-10
Missouri Mesonet^34^	37	6	31	2020-02-27
NICE Net^37^	19	6	13	2019-05-10
Oklahoma Mesonet^38^	120	64	56	2019-10-17
SCAN^39^	56	9	47	2021-04-17
USCRN	24	2	22	2020-03-20
WACNet^47^	17	3	14	2021-01-10
Other^‡^	90	58	32	2021-01-20
Grand Total	1,078	285	793	

Network	Data access	Data restrictions	Data Policy
AgriMet, Columbia-Pacific Northwest Region^27^	Publicly available		
AgriMet, Missouri Basin Region and Arkansas-Rio Grande-Texas Gulf Regions^27^	Publicly available		
AZMET^28^	Publicly available		https://azmet.arizona.edu/about
CIMIS^26^	Publicly available		
CoAgMET	Publicly available		
GAEMN^32^	Personal communication via email	Access requires contacting the network. Data provided on the condition that the network be cited.	
HPRCC^33^^,†^	Personal communication via email	Can be reproduced with current publicly available web application programming interface (API). Historical data provided on the condition that the network being cited in paper. We have also cited the networks for which the HPRCC acts as an aggregator^†^.	
Missouri Mesonet^34^	Publicly available		
NICE Net^37^	Publicly available		
Oklahoma Mesonet^38^	Publicly available	Data are publicly available; publication of data requires paying data fees. We obtained permission to publish these data with waived fees via an in-person conversation.	https://mesonet.org/about/terms-of-use
SCAN^39^	Publicly available		
USCRN	Publicly available		
WACNet^47^	Publicly available		
Enviroweather	Publicly available	Right to use is given for academic and research purposes.	https://enviroweather.msu.edu/TermsOfAgreement
FAWN	Publicly available		
West Texas Mesonet	Paid access through Synoptic (https://synopticdata.com/)	Permission to publish data obtained via written communication.	https://www.mesonet.ttu.edu/disclaimer
WRCC	Publicly available		
ZiaMet	Personal communication via email	Can be reproduced with current publicly available data request form.	
UCC	Publicly available		https://climate.usu.edu/policy.php
USDA-ARS	Publicly available		https://www.ars.usda.gov/southeast-area/stuttgart-ar/dale-bumpers-national-rice-research-center/docs/weather-stations/

^†^Includes stations from HPRCC, Iowa Environmental Mesonet, Kansas Mesonet, NDAWN, Nebraska Mesonet, and South Dakota Mesonet. ^‡^Includes stations from Enviroweather, FAWN, West Texas Mesonet, WRCC, New Mexico’s ZiaMet, UCC, and USDA-ARS.

Weather data are collected for a variety of reasons for a variety of stakeholders and not all weather stations are suitable for reference ET^19,50^. A mesonet is a network of automated, fixed, surface weather observing stations with a spatial density of ~one station per 1000 km^2^ that monitors environmental variables in the vertical domain between 10 m above and 1 m below ground surface and provides high data quality and reliable near-real time weather data^51^. Such stations have the specific objective of collecting observations that are representative of the mesoscale environment on the scale of 3–100 km, whereas an agricultural meteorological station provides detailed information on the very lowest layer of the atmosphere that may include soil temperature, soil moisture, and ETo^52,53^. As such, many stations are not sited specifically within the microclimate of the agricultural footprint for the accurate calculation of ET^19,50^.

### Data processing and quality control

The *agweather-qaqc* package^23^ was used for data visualization, screening, automated and manual QC, and calculation of reference ET. It includes modules to ingest data from tabular files (e.g., CSV) and a corresponding configuration file, which specifies station metadata (e.g., latitude, longitude, elevation, anemometer height), weather variables, and respective units within the input data file. *agweather-qaqc* was developed to handle most common input weather variables, units, and data formats, standardizing variables, QC, and calculations for consistency and reproducibility as described below.

### Data Pre-processing

Several pre-processing steps were performed using *agweather-qaqc*: (1) raw data were read and variable names were standardized across all station files, (2) meteorological variables were converted into units compliant with ASCE-EWRI standardized reference ET calculations (e.g., air temperature to °C, solar radiation to MJ m^−^², vapor pressure to kPa, wind speed to m s^−^¹), and (3) data were systematically screened and removed due to physical limits and obvious erroneous values. Examples include negative precipitation, negative wind speed, daily total solar radiation values at or near zero during expected daylight hours, or air temperature readings that fall outside extreme, historically plausible ranges for the station’s location (see the Technical Validation section).

### Data quality control

The QC procedures applied to solar radiation, air temperature, humidity, and wind speed were applied based on the established guidelines described below.

#### Solar radiation (Rs)

For each day, theoretical clear sky solar radiation, ${{\rm{R}}}_{{\rm{so}}}$, was calculated following ASCE-EWRI guidelines. ${{\rm{R}}}_{{\rm{so}}}$ represents the theoretical maximum daily incoming solar radiation a station received under clear sky conditions, based on station latitude, elevation, day of the year, and atmospheric water vapor content (derived from humidity data). ${{\rm{R}}}_{{\rm{s}}}$ data were compared to ${{\rm{R}}}_{{\rm{so}}}$ and values that significantly exceed ${{\rm{R}}}_{{\rm{so}}}$ (commonly due to data logger or sensor electrical issues) were removed. Along with progressive calibration drift, solar radiation sensors are subject to maintenance issues related to the sensor being out-of-level and dust or debris on the optics. To identify and correct ${{\rm{R}}}_{{\rm{s}}}$ drift or anomalously low values due to temporary obstructions, ${{\rm{R}}}_{{\rm{s}}}$ was systematically compared to ${{\rm{R}}}_{{\rm{so}}}$ and adjusted based on the ratio of ${{\rm{R}}}_{{\rm{s}}}$ to ${{\rm{R}}}_{{\rm{so}}}$. The ${{\rm{R}}}_{{\rm{s}}}$ record was divided into 60-day periods, and a percentile correction factor (${\rm{C}}{{\rm{F}}}_{{\rm{P}}}$) was calculated and applied to all ${{\rm{R}}}_{{\rm{s}}}$ data within each period based on the assumption that observed ${{\rm{R}}}_{{\rm{s}}}$ should approach ${{\rm{R}}}_{{\rm{so}}}$ on the clearest days. For each 60-day period, ${\rm{C}}{{\rm{F}}}_{{\rm{P}}}$ was calculated as the ratio of the average ${{\rm{R}}}_{{\rm{so}}}$ to the average ${{\rm{R}}}_{{\rm{s}}}$ as:

$${\rm{C}}{{\rm{F}}}_{{\rm{P}}}=\frac{\bar{{{\rm{R}}}_{{\rm{so}}}{\rm{P}}}}{\bar{{{\rm{R}}}_{{\rm{s}}}{\rm{P}}}}$$

where $\bar{{{\rm{R}}}_{{\rm{so}}}{\rm{P}}}$ and $\bar{{{\rm{R}}}_{{\rm{s}}}{\rm{P}}}$ are the average ${{\rm{R}}}_{{\rm{so}}}$ and ${{\rm{R}}}_{{\rm{s}}}$ values, respectively, for the selected clearest days in period ${\rm{P}}$. For the CONUS-AgWeather dataset, respective ${{\rm{R}}}_{{\rm{s}}}$ and ${{\rm{R}}}_{{\rm{so}}}$ data within top 10^th^ percentile of a 60-day period (i.e., 6 days) were selected and used to compute ${\rm{C}}{{\rm{F}}}_{{\rm{P}}}$. This 60-day period was used to account for seasonal variations at the stations while remaining responsive to potential sensor drift. ${\rm{C}}{{\rm{F}}}_{{\rm{P}}}$ values were then multiplied by all ${{\rm{R}}}_{{\rm{s}}}$ values within each respective 60-day period. If ${\rm{C}}{{\rm{F}}}_{{\rm{P}}}$ ranged from 0.97 to 1.03, no adjustment was applied, and if ${\rm{C}}{{\rm{F}}}_{{\rm{P}}}$ was <0.5 or >1.5, ${{\rm{R}}}_{{\rm{s}}}$ data for that period were deemed erroneous and were removed. It is expected that a cloud-free day should occur with some regularity for areas with significant agriculture, especially in semi-arid and arid areas of the Western U.S.^23^. However, the 60-day period may need to be smaller in more humid regions and during winter months.

#### Air temperature (${T}_{\max }$, ${T}_{\min }$)

Temperature data were quality controlled using a modified Z-score approach, as detailed by Iglewicz and Hoaglin (1993). This method is minimally influenced by outliers, particularly in smaller samples, as it utilizes the median and median absolute deviation (MAD), i.e., ${\rm{media}}{{\rm{n}}}_{{\rm{i}}}\left({{\rm{x}}}_{{\rm{i}}}-\widetilde{{\rm{x}}}\right)$, where $\widetilde{{\rm{x}}}$ is the median of the sample observations ${{\rm{x}}}_{{\rm{i}}}$. The modified Z-score (${{\rm{M}}}_{{\rm{i}}}$) for each observation ${{\rm{x}}}_{{\rm{i}}}$ was then computed as:

$${{\rm{M}}}_{{\rm{i}}}=\frac{0.6745\left({{\rm{x}}}_{{\rm{i}}}-\widetilde{{\rm{x}}}\right)}{{\rm{MAD}}}$$

Here, observations with $|{{\rm{M}}}_{{\rm{i}}}|$ > 3.5 were flagged as potential outliers and removed, as recommended by Iglewicz and Hoaglin (1993).

#### Relative humidity ($R{H}_{\max }$, $R{H}_{\min }$)

Daily ${\rm{R}}{{\rm{H}}}_{\max }$ should approach or reach 100% on at least a few days a year, usually coinciding with precipitation events, early morning condensation or dew, or periods of high atmospheric moisture due to irrigation-driven increases in ET^1,21^. This is particularly true for an agricultural weather station to meet the requirements of reference ET; however, less expensive capacitive hygrometers common to most networks lose accuracy above 95%^54,55^. To correct for sensor drift and inaccuracies, a yearly correction factor (${\rm{C}}{{\rm{F}}}_{{\rm{Y}}}=100/\bar{{\rm{R}}{{\rm{H}}}_{\max {\rm{Y}}}}$) was calculated, where $\bar{{\rm{R}}{{\rm{H}}}_{\max {\rm{Y}}}}$ is the average of the top 1%, or the 3 highest values if sufficient data exist, of the highest ${\rm{R}}{{\rm{H}}}_{\max }$ values within year ${\rm{Y}}$. This factor was derived by comparing the topmost percentile of observed ${\rm{R}}{{\rm{H}}}_{\max }$ values within a calendar year to the expected 100%. CF_Y_ was then applied to all ${\rm{R}}{{\rm{H}}}_{\max }$ and ${\rm{R}}{{\rm{H}}}_{\min }$ observations for that year. It should be noted that the CF_Y_ also adjusts the observed values down to be in line with the expected 100% when the sensor has a high bias over 100%. The number of points used for this calculation was adjusted for years with incomplete data records.

#### Wind speed (${u}_{Z}$)

If wind speed (${{\rm{u}}}_{{\rm{Z}}}$) was measured at an anemometer height (${\rm{Z}}$, in meters) other than the standard 2-meter reference height, it was adjusted to ${{\rm{u}}}_{2{\rm{m}}}$ using the logarithmic wind profile equation as specified by ASCE-EWRI (2005)^24^:

$${{\rm{u}}}_{2{\rm{m}}}={{\rm{u}}}_{{\rm{Z}}}\frac{4.87}{\mathrm{ln}\left(67.8{\rm{Z}}-5.42\right)}$$

The primary QC for wind speed relied on manual inspection of the interactive time series plots generated by *agweather-qaqc*. We investigated wind speed patterns, such as trends or rapid changes in wind speed (e.g., due to failing anemometer bearings, nearby obstructions, tree growth, etc.), prolonged periods of zero or constant wind speed, or values that appear implausibly high or low relative to the typical wind regime of the station or nearby stations. Such identified periods were flagged and removed.

#### Precipitation

Precipitation data were screened for erroneous values (e.g., negative values) and extremely large events. We removed daily precipitation values greater than 610 mm, approximately one-third of the world-record 24-hour precipitation total of 1,828 mm.

### Gap-Filling

Gap-filling was not performed in the CONUS-AgWeather dataset beyond creating a complete record of ${{\rm{R}}}_{{\rm{so}}}$ to support the QC of observed ${{\rm{R}}}_{{\rm{s}}}$. The *agweather-qaqc* package incorporates optional routines for gap-filling missing data in the daily records^23^, but these were not applied to the CONUS-AgWeather dataset. When applying the dataset for any application that involves temporal aggregation (cumulative, averaging, etc.) it is important that users consider and report the gaps if they choose not to gap-fill the data.

### Calculation of reference evapotranspiration

**Fig. 3:**
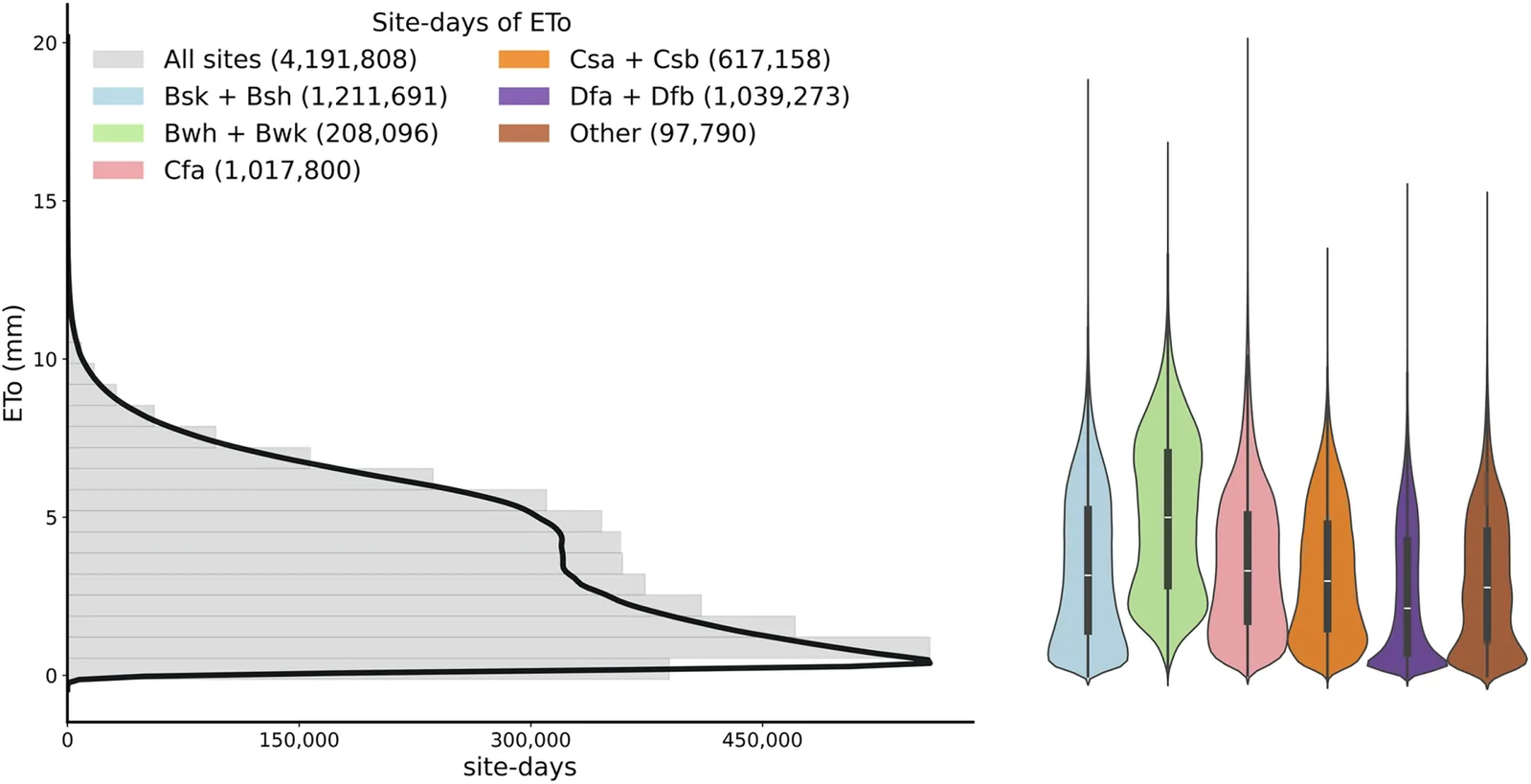
Histogram of daily ETo in the CONUS-AgWeather dataset with corresponding violin plot distributions across major KG climate zones^25^: cold and hot semi-arid steppe (Bsk + Bsh); hot and cold desert (Bwh + Bwk); humid subtropical (Cfa); hot- and warm-summer Mediterranean (Csa + Csb); and hot- and warm-summer humid continental (Dfa + Dfb). ‘Other’ includes all other climate zones outside the five major zones listed. Corresponding plots for all CONUS-AgWeather variables are available on Zenodo (10.5281/zenodo.18673483).

Following the comprehensive QC, the daily grass (i.e., short) reference ET (ETo) and alfalfa (tall) reference ET (ETr) were calculated according to the ASCE Standardized Penman-Monteith (ASCE-PM) equation using *RefET* Python library. For stations where vapor pressure was not available directly, it was derived from dew point temperature or relative humidity data present in the record following recommendations and guidelines of ASCE-EWRI (2005). A histogram of daily ETo data for all 793 stations, and violin plots of ETo by KG climate zones^25^, is illustrated in Fig. 3.

## Output Generation and Data Archiving

The CONUS-AgWeather dataset includes comprehensive outputs, including processing log files, interactive time series plots, and tabular data files, as described below:

- Log Files: Detailed, human-readable log files for each station. These files record all automated QC checks, user-initiated corrections, and parameters used for adjustments of data during the processing.
- Interactive Plots: Interactive time series plots (rendered using Bokeh) that display both the pre-QC (original) and post-QC (corrected) data for solar radiation, air temperature, humidity, wind speed, and precipitation. These plots, saved as standalone HTML files for easy sharing and archiving, are invaluable for visual assessment of data quality, the impact of applied corrections, and for facilitating manual review.
- Data Files: The primary output for each station consists of structured data files (Microsoft Excel spreadsheets and parquet files) containing the fully quality-controlled observations and the numerical difference (delta values) between original and corrected data where adjustments were made.

In summary, CONUS-AgWeather output is a collection of individual station log files, interactive plots, and post-QC data files for all 793 weather stations, in the format text files, Bokeh HTML, and both Microsoft Excel spreadsheets and parquet files, respectively.

## Data Records

Each station record within the CONUS-AgWeather dataset includes the following 23 standardized variables, with the ones in bold font used in the ASCE-PM reference ET^24^ calculation:

1. Station ID: Unique identifier for the weather station (<Number>_<State abbreviation>, e.g., 001_AR).
2. Latitude: Latitude of the weather station in decimal degrees (0.0001°). Some networks report at a reduced precision to protect the privacy of the landowner.
3. Longitude: Longitude of the weather station in decimal degrees (0.0001°). Some networks report at a reduced precision to protect the privacy of the landowner.

For weather stations reporting reduced coordinate precision (in degrees, minutes, seconds or DMS), the “Coordinate Precision” column of the metadata file notes “Lat/Long coordinates translated from DM, no DMS available.” This column value is kept empty for all other stations.

4) Elevation: Elevation of the weather station in meters.
5) Date: Observation date (YYYY-MM-DD).
6) **TMax**: Daily maximum air temperature (°C).
7) **TAvg**: Daily average air temperature (°C), computed as the average of the daily maximum and minimum temperatures.
8) **TMin**: Daily minimum air temperature (°C).
9) **Ea**: Daily mean vapor pressure (kPa), either directly from the QC’d record or calculated using *agweather-qaqc*. If Ea was calculated, *agweather-qaqc* used only the most preferred form of humidity data available as specified in the ASCE-EWRI standard^24^. Note that preferred humidity data was QC’d prior to calculation of Ea if Ea was not provided directly (e.g., when only RHMax and RHMin are provided in the observational record).
10) TDew: Daily average dew point temperature (°C), either from the QC’d record or calculated.
11) RHMax: Daily maximum relative humidity (%). This variable may not be present if it is not provided by the weather station network. RHMax is used in the ASCE-PM reference ET^24^ calculation only if Ea or TDew are not provided in the observational record.
12) RHAvg: Daily average relative humidity (%). This variable may not be present if it is not provided by the weather station network. RHAvg is only used in the ASCE-PM reference ET^24^ calculation if no other sources of humidity observations are provided in the record.
13) RHMin: Daily minimum relative humidity (%). This variable may not be present if it is not provided by the weather station network. RHMin is used in the ASCE-PM reference ET^24^ calculation only if measured Ea or TDew are not provided in the observational record.
14) Compiled Ea: Daily mean vapor pressure (kPa). This is an aggregate record that uses all forms of humidity data present, not just the most preferred, to calculate as complete a record of Rso as possible for the QC of Rs.
15) **Rs**: Average daily incoming shortwave solar radiation (W m^−^²).
16) Optimized TR Rs: Optimized Thornton-Running solar radiation (W m^−^²), which computes a modeled version of incoming shortwave radiation according to Thornton and Running’s equation. This variable is calculated to provide the option of a full record of solar radiation but is separate from QC’d observations of solar radiation.
17) **Rso**: Computed clear-sky solar radiation (W m^−^²) for QC of observed Rs. The values are computed using station latitude and equations that model the effects of precipitable water in the atmosphere on incoming solar radiation, as described in the ASCE-EWRI (2005)^24^.
18) Measured Uz: Daily average wind speed at the actual height of the anemometer (m s^−^¹).
19) Anemometer Height: Height of the anemometer at the station (m).
20) **Uz at 2 m**: Daily average wind speed adjusted to 2 m height (m s^−^¹).
21) Precipitation: Daily total precipitation (mm).
22) ETo: Daily grass reference ET (mm).
23) ETr: Daily alfalfa reference ET (mm).

## Data Format and Availability

The CONUS-AgWeather dataset^56^ is available as a zip file (‘CONUS-AgWeather_v1.zip’), containing Microsoft Excel workbooks (.xlsx) and parquet (.parquet) files for individual stations (10.5281/zenodo.18122156). Each Excel workbook contains two spreadsheets or tabs: ‘Corrected Data’ (corrected records) and ‘Delta (Corr - Orig)’ (difference between corrected and original data). Accordingly, there are two parquet files corresponding to the two Excel spreadsheets for each station, e.g., ‘001_AR_data_corrected.parquet’ and ‘001_AR_data_delta.parquet.’ Detailed metadata with station-specific QC notes, human readable plaintext log files, interactive HTML Bokeh^57^ plots of the pre-and post- QC datasets, and station location maps (like Fig. 2; 600 DPI png files) and statistics (csv file) for all variables are also provided. The period of record for each station varies, and this information is included in the metadata.

## Technical Validation

The technical quality and reliability of the CONUS-AgWeather dataset was established through the systematic, standardized, and reproducible QC procedures within the *agweather-qaqc* Python package ^23^. Visual inspection of individual site locations using satellite imagery to confirm station-siting in agricultural land was the first hurdle, then weather data time series further enhanced the validation of the final dataset.

After the initial ingestion of weather station data, the records were carefully assessed for key variables required to compute the ASCE-PM reference ET^24^. These variables included air temperature, solar radiation, wind speed, and humidity. Each weather station was evaluated based on its availability of data for all four variables. Stations that did not have at least two years of continuous, quality data during the growing season for all four variables were removed, resulting in a reduction of 21 stations from the initial dataset of 1,078 stations.

**Fig. 4:**
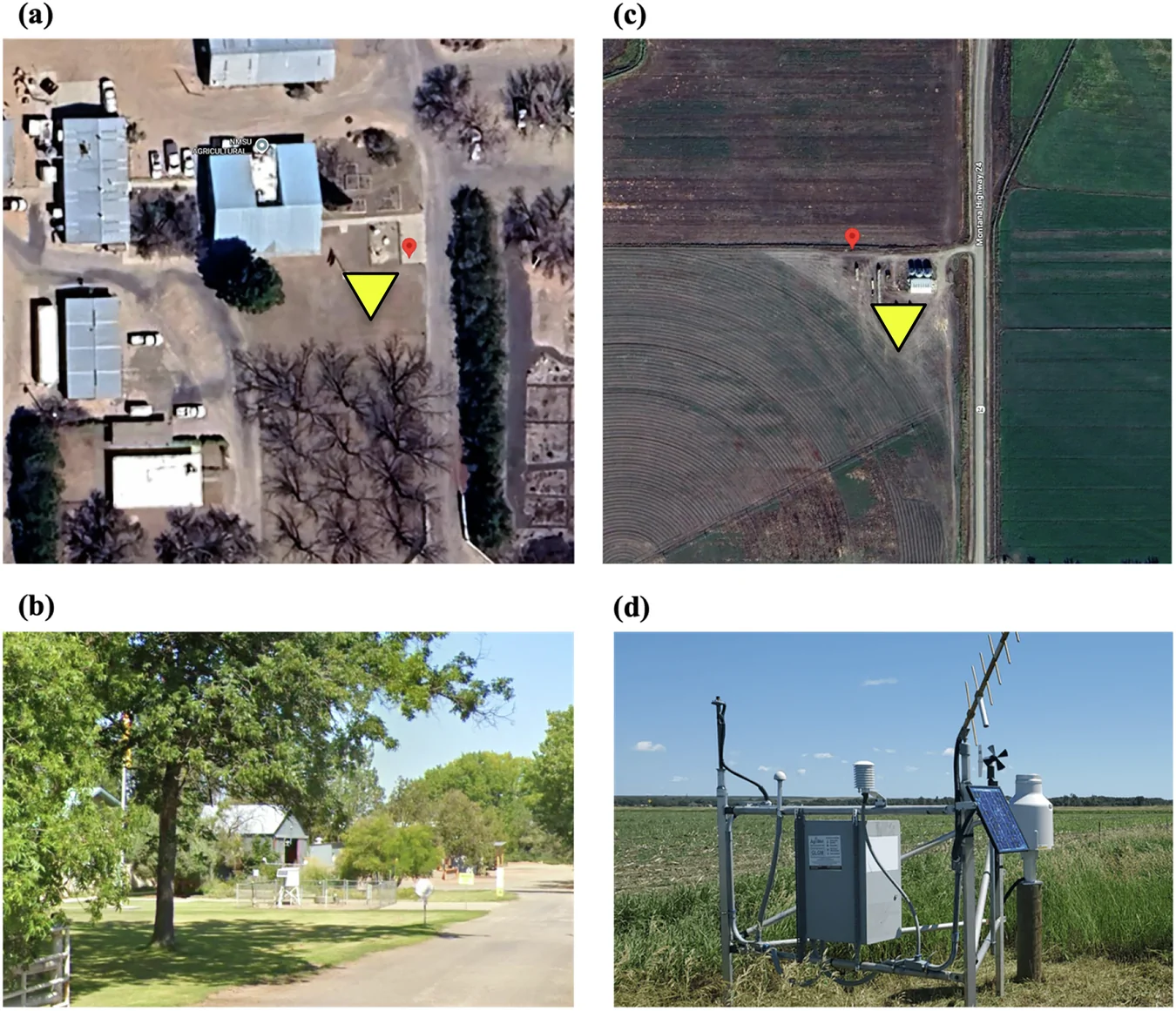
(**a**) show satellite and **(b)** street views for a ZiaMet^44^ station in Los Lunas, New Mexico that was removed from the CONUS-AgWeather dataset because of poor station siting with many obstructions (e.g., trees) affecting measurements. In contrast, **(c)-(d)** show U.S. Bureau of Reclamation AgriMet^27^ station (465_MT) in Glasgow, Montana, that is well-sited in an area surrounded by agriculture, with the nearest structures being greater than 76 m (250 ft) away.

We visually inspected the environment surrounding each weather using current and historical imagery from Google Earth and Google Street View, to ensure the station was located within well-watered agricultural areas, per recommendations and guidelines of ASCE-EWRI (2005)^24^ (Fig. 4a–d). Poor station siting was carefully considered, specifically stations located in urban or non-agricultural areas, and those possibly affected by obstructions (e.g., trees, buildings) and microclimates (e.g., water bodies, barren areas, urban heat), were flagged and removed. Stations with a potential wind obstruction within 15 m (50 ft) were excluded, but a subjective determination on minimum distance was made for larger barriers, such as a stand of trees or large buildings.

Location and visual screening resulted in a further reduction of 264 stations, i.e., a total of 285 stations were removed from the initial dataset of 1,078 stations. Our screening criteria are subjective and no strict, quantifiable spatial threshold (e.g., minimum distance to structures) was mathematically defined. Figure 1 and Fig. 2 illustrate the spatial distribution of initial (1,078 stations) and final (793 stations) CONUS-AgWeather data, respectively.

Technical validation of automated statistical QC using *agweather-qaqc*^23^ (detailed in the Methods section) corrected or flagged suspicious time series data. Manual visual inspection of flagged data identified any trends or abrupt shifts related to sensor malfunction or data logging errors. Stations with frequent, suspect, or unresolved data quality issues were removed from the dataset.

**Fig. 5:**
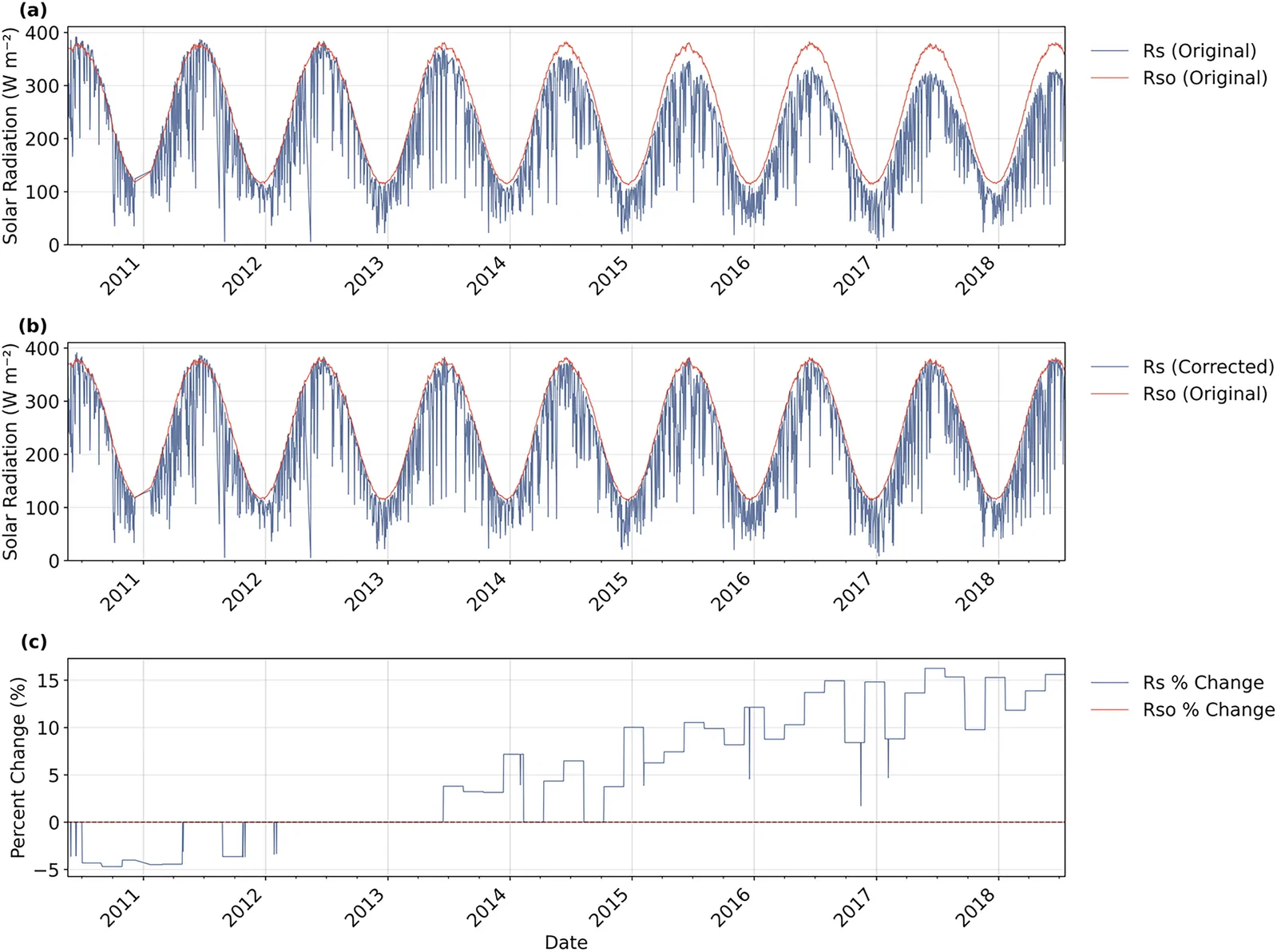
**(a)** Original daily shortwave radiation (Rs) measurements and computed clear-sky solar radiation (Rso) at a NICE Net^37^ station (635_NV) located in Reno, Nevada, illustrating consistent sensor drift beginning in summer of 2014. **(b)** Corrected daily Rs after QC adjustment using Rso as a limit. **(c)** Percent change between the original (pre-QC) and corrected (post-QC) Rs values for each sixty-day period (i.e., ((corrected - original) / original) * 100)^23^. Note that Rso is not corrected.

Common errors in measured Rs from pyranometers include calibration drift, improper leveling, sensor degradation, or temporary obstructions from dust and debris^22,58^. Measured Rs should approach or slightly exceed the theoretical clear-sky solar radiation (Rso) at least a few days a season, particularly in the western U.S. For example, the measured maximum daily Rs frequently approaches Rso across all seasons at a NICE Net station in Reno, NV, as expected for this arid site location, but begins to drift consistently lower than Rso in summer of 2014 (Fig. 5a). From 2014 onward, maximum daily Rs was consistently lower than Rso, indicating sensor drift that was corrected to measured Rs during the QC process (Fig. 5b) using 60-day correction factors (Fig. 5c).

As part of the technical validation process, time series plots of pre- and post-corrected Rs as well as percent change values, as illustrated in Fig. 5, were visually inspected for every station to ensure corrections were justified and sensible. Automated corrections to Rs made with *agweather-qaqc* are based on manual Rs corrections^20,24,58^; however, *agweather-qaqc* is less subjective, more reproducible, and fully documented in the CONUS-AgWeather dataset.

**Fig. 6:**
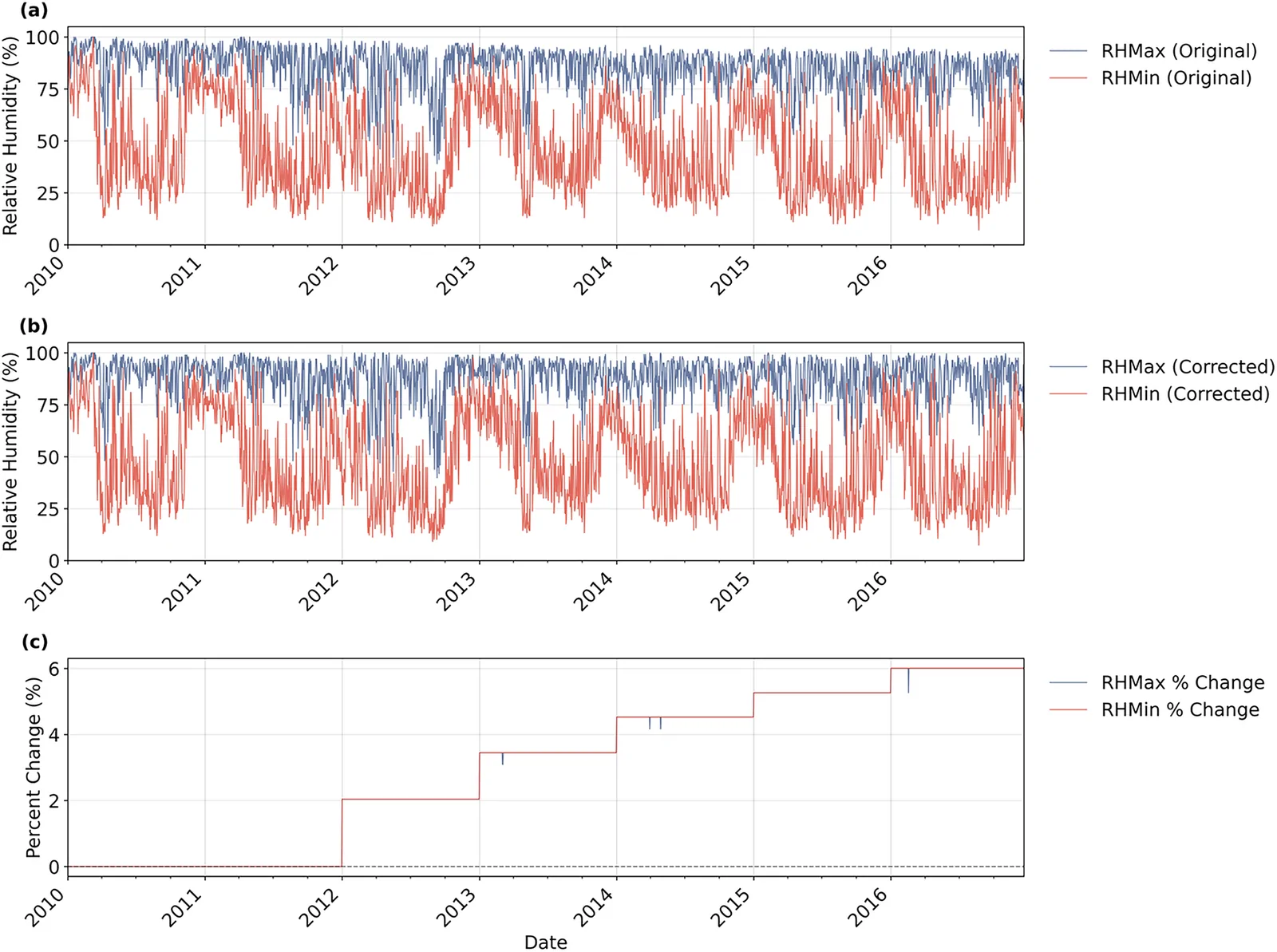
**(a)** Daily relative humidity maximum (RHMax) and minimum (RHMin) from a SCAN^39^ station (1069_MT) in Sidney, Montana with pronounced sensor drift, (**b)** corrected data after a year-based percentile adjustments, and **(c)** the percent change for both.

Capacitive humidity-sensing elements, common in combined air temperature and humidity sensors, drift over time and should be replaced every two to three years; however, that is often not practical in network operations. As shown in Fig. 6a, measured daily maximum relative humidity (RHMax) shows a clear trend starting near 100% and decreasing to ~80% over 7 years. RHMin is also decreasing through time at a similar rate. Figure 6c shows the percent change in pre- and post-corrected RH as a result of yearly correction factors computed and applied by *agweather-qaqc* to both RHMax and RHMin, resulting in RH data that are within expected limits and are free from drift artifacts (Fig. 6b).

**Fig. 7:**
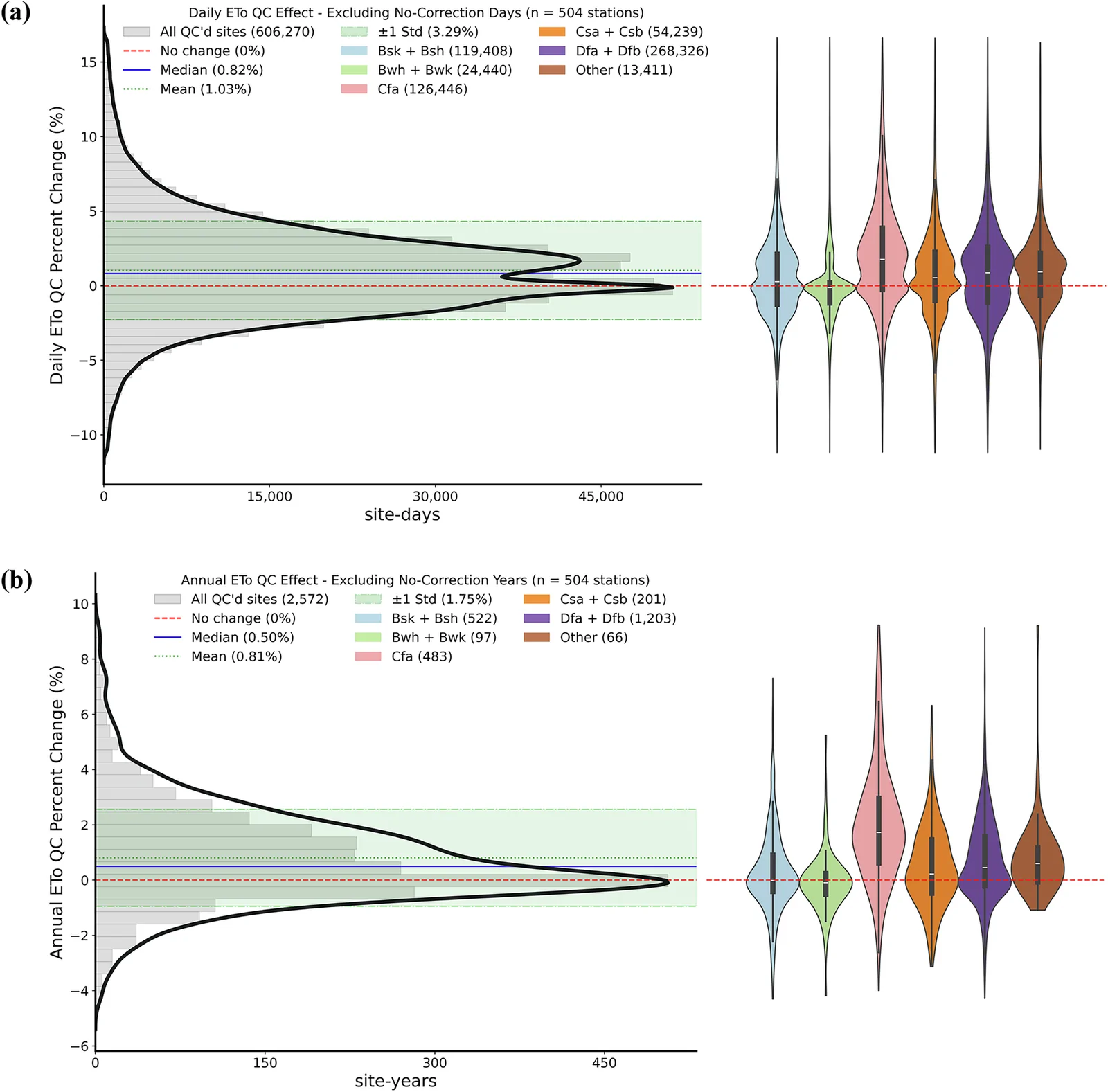
Distributions of **(a)** daily and **(b)** annual ETo change due to QC (in % change) across major KG climate zones^25^ based on 504 (out of 793) CONUS-AgWeather stations with complete annual records. Left panels show histograms and kernel density estimates; right panels show violin plots stratified by KG climate zones: cold and hot semi-arid steppe (Bsk + Bsh); hot and cold desert (Bwh + Bwk); humid subtropical (Cfa); hot- and warm-summer Mediterranean (Csa + Csb); and hot- and warm-summer humid continental (Dfa + Dfb). ‘Other’ includes all other climate zones outside the five major zones listed. Reference lines indicate no change (0%, red dashed), median (blue solid), mean (green dotted), and ±1 standard deviation (green shaded region). Data are trimmed to the 1^st^–99^th^ percentile range for visualization, while sample sizes reflect all QC-corrected records prior to trimming.
  In addition, Fig. 7 reveals modest variations in corrected ETo across KG climate zones^25^, though all climate classes show positive and negative corrections centered near zero. Stations located in humid subtropical (Cfa) regions exhibited the highest variability and largest positive QC corrections, with a mean daily percent change in ETo due to QC of +2.02 ± 3.61% and annual change of +2.00 ± 2.15%. In contrast, stations in arid desert climates (Bwh + Bwk) showed minimal QC effects with near-zero mean corrections and the lowest variability (daily: +0.08 ± 3.00%, annual: −0.09 ± 1.13%), indicating more stable measurement conditions in these environments.
  Results demonstrate that while individual daily QC corrections can be substantial, their cumulative annual impact on ETo estimates are relatively modest, typically within ±2–3% for most stations. This indicates that supporting the robustness of the QC methodology generally preserves the majority of the original observations while correcting for sensor drift, data gaps, and measurement anomalies^20,58^.
- Achieving Benchmark Quality: The overarching goal of the comprehensive QC process was to produce a dataset of benchmark quality. This benchmark dataset can serve as a reliable reference for developing, validating, and bias correcting ET and meteorological products (e.g., satellite-derived ET datasets, gridded weather data, and hydrologic models), for calculating accurate crop and irrigation water requirements, and for integration within water resource decision making processes. For example, the OpenET Consortium relies on the CONUS-AgWeather dataset for bias correcting gridMET-based ETo^7^, which is a key input to the majority of OpenET models^15,16^. Based on the work of Volk *et al*.^62^ that use 79 independent flux stations^14^ throughout the CONUS, gridMET ETo bias correction using the CONUS-AgWeather dataset improves the accuracy of monthly ETo for cropland sites, and also forests, shrublands, grasslands, and wetlands (Fig. 8).

As described and illustrated above, manual site location and data time series inspections, along with the automated QC procedures using *agweather-qaqc* are foundational to the CONUS-AgWeather dataset and achieving benchmark data quality. Our technical validation approach focused on adherence to scientific best practices based on standardized methods and guidelines. The following elements summarize our technical validation:

- Standardized and Documented Procedures: The application of consistent and standardized QC rules and algorithms across all stations and variables, based on widely accepted meteorological and agricultural engineering principles^20,24,59^. All QC procedures are documented and reproducible within the *agweather-qaqc* package^23^.
- Physically-Constrained Corrections: Adjustments are constrained by physical limits, expected values, and well-established practices in the meteorological domain^20,58^. For example, Rs corrections are calculated and limited by Rso, and RH adjustments consider the likelihood of relative water vapor saturation in well-watered reference crop conditions^20,58^.
- Robust Statistical Outlier Detection: The use of physical limits for Rs and RHMax/RHMin, and non-parametric outlier detection methods, such as the modified Z-score for temperature data based on median absolute deviation about the sample median (i.e., MAD), ensuring minimal influence of outliers when statistically identifying and removal of outliers^60,61^.
- Visual Inspection and Expert Review: Remote visual inspection of site locations based on street view, aerial and satellite imagery (Fig. 4), and generation of interactive time series plots for visualization and expert review are a cornerstone of the QC process and *agweather-qaqc* output. These images and plots allow for expert review, which is crucial for identifying subtle data quality issues that automated algorithms often miss (e.g., anomalously low humidity for agricultural conditions, local obstructions affecting wind speed and precipitation) and for verifying automated corrections (Fig. 5 and Fig. 6).
- Quantifiable Impact of QC: The necessity and impact of QC steps and corrections can be substantial. For instance, uncorrected Rs (Fig. 5a) directly impacts reference ET calculations. Comparing ETo data values between the original and QC’d observations can result in substantial differences, potentially by a factor (QC / original) of only ~ 0.03 (station: 814_SD, 1992-01-07) to ~581.84 (station: 956_WY, 2017-04-29) and ~ 0.76 (station: 824_SD, 1984) to ~ 1.28 (station: 014_AZ, 2014) for daily and annual time steps, respectively, with the QC’d values being higher or lower than the original data depending on the underlying cause^20,58^.
  While the above factors represent the extreme ends of the daily and annual QC / original ETo distributions, Fig. 7 illustrates (1^st^–99^th^ percentile) the ETo change across 504 (out of 793) CONUS-AgWeather stations with complete annual records, where QC was applied (i.e., post-QC / pre-QC ≠ 1). QC corrections resulted in a median increase of ~ 0.8% in daily ETo values, with a mean increase of ~ 1%, indicating that the original station data tended to slightly underestimate ETo prior to correction. The distribution is slightly right skewed, with most corrections falling within ±5% of the original values. Notably, ~ 36% of daily observations (345,495) required no correction (i.e., post-QC / pre-QC = 1), while the remaining ~ 64% of site-days (606,270) showed measurable QC adjustments as highlighted in Fig. 7a. At the annual scale (Fig. 7b), the aggregated effect of daily corrections is dampened, with a median percent change of ~ 0.5% and mean of ~ 0.8%, as positive and negative daily corrections offset each other over the year.

**Fig. 8:**
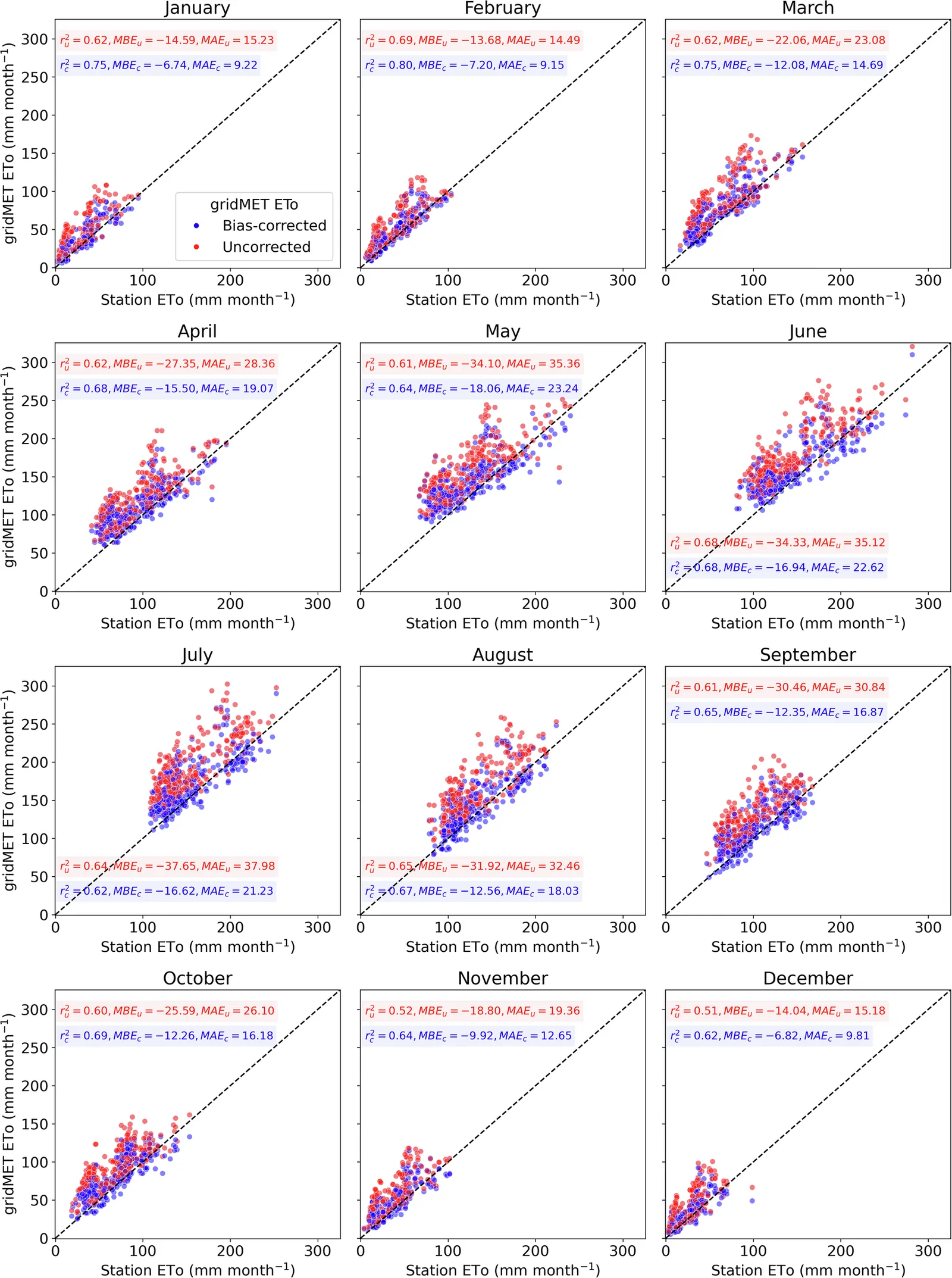
The CONUS-AgWeather dataset was validated using independent measurements from 79 flux stations^14^ and used to bias-correct gridMET^62^. Scatter plots comparing monthly uncorrected and bias-corrected gridMET ETo reveal substantial improvements in error metrics across all major land covers. Error metrics include coefficient of determination (r^2^), mean bias error (MBE), and mean absolute error (MAE). Here, the subscripts ‘u’ and ‘c’ (e.g., MBE_u_ and MBE_c_) denote uncorrected and bias-corrected ETo, respectively. This evaluation demonstrates the application of the CONUS-AgWeather dataset for bias-correcting gridded meteorological products used in operational workflows, such as OpenET^15,16,62^.

## Usage Notes

The CONUS-AgWeather dataset is intended to be a valuable resource for a diverse community of users, including agricultural engineers, hydrologists, meteorologists, water resource managers, farmers, and other users (e.g., educators). CONUS-AgWeather is a static dataset from 1981 to 2020. We plan to expand eastward, incorporate more weather stations, improve the site screening algorithm to assess site aridity, and update time series through more current years.

### Potential applications in crop management

(a) Irrigation Scheduling: Providing high quality, bias-corrected weather and reference ET information as an input for irrigation scheduling tools, leading to more precise water application and improved water use efficiency^63^, (b) Crop Water Use and Stress Assessment: Using high quality, bias-corrected weather and reference ET information in conjunction with crop models or remote sensing data to assess and monitor crop water requirements, water use, and water stress^9,10,13,64^, (c) Yield Forecasting: Supplying quality-controlled weather inputs for crop growth and yield forecasting models^65^, (d) Development and Validation of Decision Support Systems: Serving as a benchmark dataset for developing, calibrating, and validating new agricultural decision support tools^66^, (e) Climate Impact Studies on Agriculture: Providing quality-controlled historical weather data for analyzing the impacts of climate variability and change on crop production and water demand^67,68^, and (f) Pest and Disease Modeling: Use of accurate weather data for predicting the development and spread of crop pests and diseases^69^.

### General meteorological and hydrological applications

These include (a) validation and bias correction of numerical weather forecasts^70–73^, reanalysis products,^8,74^, (b) drought monitoring and assessment^75–78^, (c) surface energy balance and ET studies^16^, and (d) surface water and groundwater modeling, water resource investigations, and national scale water use reporting^5,17,79–81^.

### Limitations and considerations for users

- Weather Station Networks: Weather station networks present in the CONUS-AgWeather dataset reflect the strategic priorities of the OpenET Phase I project^16^, which focused primarily on developing an operational actual ET product in the Western U.S. Consequently, the dataset exhibits a substantially higher station density in the West compared to the East (Figs. 1,2). While we sought broader coverage, many networks, particularly in the Eastern U.S. (e.g., North Carolina ECONet^82^, Alabama Mesonet^83^, New York State Mesonet^84^, and others) were excluded to align with the Phase I timeline and specific modeling objectives. We omitted data sources that were paywalled, unavailable prior to 2020, or inaccessible during the project’s operational window. Furthermore, because the dataset was specifically created to bias-correct gridMET ETo for the OpenET actual ET models^62^, we enforced spatial filtering to exclude stations located outside of well-watered agricultural areas, ensuring that only data representative of agricultural microclimates was included in the dataset. Therefore, users focusing on the Eastern U.S. may need to supplement CONUS-AgWeather with additional local or regional meteorological networks that were either inaccessible at the time of creation or did not strictly meet the bias-correction criteria adopted in the OpenET project.
- Metadata Reliance: Some QC steps, calculation assumptions (e.g., standardized wind speed adjustment to 2 m height based on reported anemometer height), depend on the accuracy of the station metadata provided by the networks (i.e., latitude, longitude, elevation, anemometer height). Users should consult the accompanying metadata for detailed site-specific information. It should be noted that the metadata lists 20 weather station networks instead of the initial 25 as the HPRCC network includes stations from HPRCC^33^, NDAWN^35^, Nebraska Mesonet^36^, South Dakota Mesonet^40^, Iowa Environmental Mesonet, and Kansas Mesonet (see Table 1).
- Station Siting: The ASCE Penman-Monteith reference ET equation^24^ is used to calculate reference ET for a hypothetical standardized reference crop surface (i.e., well-watered grass or alfalfa) and assumes that weather data is representative of reference conditions. Out-of-specification siting and deviations from reference conditions (e.g., excessive dryland within the station’s fetch, tall crops, trees, or other obstructions) can influence calculated reference ET. Extensive QC of station siting was conducted, but ground-truthing was limited to a few stations.
- Period of Record: The length of available data records varies by station but all end in 2020. Users should verify that the period of record for selected stations is adequate for their intended applications.
- Gap Handling: We recommend that users (1) calculate completeness over the exact period of interest; (2) avoid cumulative summaries when missingness exceeds an application-specific threshold; (3) fill short gaps using documented approaches such as nearby high-quality stations, network-provided data, or gridded meteorological/reference ET products adjusted to local station conditions; (4) avoid or flag long gaps, especially during periods of high evaporative demand; and (5) report the gap-filling method and uncertainty when filled data are used.

## Data Availability

The CONUS-AgWeather dataset^56^ was generated using publicly available, open-source Python packages to process and QC publicly available weather data. The primary package for quality assurance and quality control is: a) *agweather-qaqc*: The latest source code^23^, documentation, and all files required to demonstrate example usage are available on GitHub at https://github.com/WSWUP/agweather-qaqc. Although *agweather-qaqc* v0.7.0 was used to generate CONUS-AgWeather, the latest *agweather-qaqc* v1.0.4^23^ provides the same functionalities (with substantial workflow improvements) and can also be used to reproduce the same dataset. *agweather-qaqc* v0.7.0 and the station config files (.ini) are included in the Zenodo repository (10.5281/zenodo.18122156) as separate zip files, i.e., ‘agweather-qaqc-0.7.0.zip’ and ‘agweather-qaqc-0.7.0-config_files.zip’, respectively. The calculation of standardized reference evapotranspiration (ETo and ETr) was performed using: b) *RefET*: This package (Version 0.4.2), which implements the ASCE Standardized Reference Evapotranspiration Equation^24^, is available on GitHub (https://github.com/WSWUP/RefET) and PyPI (https://pypi.org/project/refet)^85^. In addition, the Python scripts used for generating the maps (Fig. 2) and plots (Fig. 3 and Figs. 5–8) are available on GitHub (https://github.com/Open-ET/gridMET-bias-correction) and Zenodo (10.5281/zenodo.18673483). The open availability of these software tools ensures transparency and reproducibility of the CONUS-AgWeather dataset generation process and allows other researchers to apply identical or adapted methodologies to their own agricultural weather datasets.
